# Phylogeography, genetic diversity and population structure in a Patagonian endemic plant

**DOI:** 10.1093/aobpla/plx017

**Published:** 2017-04-27

**Authors:** Alicia López, Marisa G. Bonasora

**Affiliations:** 1 Instituto de Botánica Darwinion (ANCEFN – CONICET), Labardén 200, B1642HYD San Isidro, Argentina; 2 Cátedra de Botánica Sistemática, Facultad de Agronomía, Universidad de Buenos Aires, Av. San Martín 4453, C1417DSE Buenos Aires, Argentina

**Keywords:** cpDNA, distribution modelling, genetic diversity, ISSR, *Oxalis*

## Abstract

Phylogeographical patterns provide valuable insight into the historical processes underlying diversification, and may provide a better understanding of biodiversity, dispersal modes, diversification times, extinctions, refuge areas and other species-/population-level processes. Here, we examine the genetic structure of *Oxalis laciniata*, a representative of *Oxalis*, which is an important emerging model in evolutionary biology and phylogenetic studies. We analyse genetic correlation, haplotype diversity and genetic structure. In this study, cpDNA reveals the presence of 16 haplotypes, connected in tree networks. Genetic diversity is high and polymorphism is low among populations based on ISSR markers. Both clustering and analysis of the structure of the population indicate two different groups. Distribution modelling predicts two potential distribution areas. Our main conclusions are: (i) The phylogeographical pattern demonstrates non-random organization of genetic variability since two distinct groups can be distinguished; (ii) two refugia are proposed: one is situated in the SE, holding the most ancestral haplotypes; and the second one is situated in the SW; (iii) we propose an *in situ* diversification hypothesis for the populations located in the steppe; (iv) the centre of diversification coincides with the centre of the distribution; (v) distribution modelling shows a strong correspondence with the distribution of the species but it also suggests the possibility of occurrence in the Central Andes.

## Teaser

Oxalis lacinata is endemic to the Patagonia region, including high mountains and steppe, and even grows on the coast. Its marked polymorphism made us think that the genetic variability behind this would explain both its morphological variability and its adaptability to different ecological niches. Based on molecular analysis, we detected non-random organization of genetic variability. We propose two refugia and we were able to estimate the diversification centre, which coincides with the centre of the distribution. Distribution modelling suggested the possibility of occurrence in the Central Andes, in addition to its known distribution.

## Introduction

Phylogeographical studies can provide a better understanding of the biodiversity, dispersal modes, diversification times, extinctions, refuge areas and other species-/population-level processes (Turchetto-Zolet *et al.* 2013). Recent reviews of the phylogeographical patterns within some specific areas in Patagonia provide valuable insight into the historical processes underlying diversification in this region (Domínguez *et al.* 2006; Morando *et al.* 2007; Jakob *et al.* 2009, 2010; Tremetsberger *et al.* 2009; Cosacov *et al.* 2010; Albino 2011; Pardiñas *et al.* 2011; Sérsic *et al.* 2011; Sede *et al.* 2012; Nicola *et al.* 2014; Morello and Sede 2016). The phylogeographical approach may also offer a means of estimating the historical processes that have influenced the structure of the genetic variation now observed in a given species (Avise 2000). Due to phylogeography is applicable to problems below and above the species boundary level, it can cover a wide range of plant evolutionary patterns (Longo *et al.* 2014).

The current geographical distribution of a species can be estimated using niche-based models that predict the presence or absence in certain places (Guisan and Thuiller 2005; Soberon and Peterson 2005). Such models can take into account potential future responses to global climate change (Roura-Pascual *et al.* 2004).

Patagonia Argentina extends from Neuquén and Río Negro provinces (delimited to the north by the Neuquén and Colorado rivers, c. 36°37′S) to Cape Horn (56°S) and it is currently partitioned into two main ecoregions. The High Andean–Patagonian ecoregion is mountainous, cold with precipitations above 300 mm per year; and the Patagonian Steppe ecoregion extends eastward to the Atlantic Ocean and is mostly low-lying, cold, dry and characterized by scattered herbs and shrubs (Cabrera and Willink 1973; León *et al.* 1998). Our species of interest, *Oxalis laciniata*, is distributed in Chubut, Santa Cruz and Tierra del Fuego e Islas del Atlántico Sur Provinces (from 45°35′38″ to 53°41′28″S and from 65°55′4″ to 72°16′4″W) and inhabits both the Patagonian ecoregions of the High Andes and the Steppe.

*Oxalis laciniata* is endemic from Patagonia region, markedly polymorphic species, which is mainly manifested in the variation in the shape and size of the leaflets. This species also has a distribution that includes the high mountains, up to 2200 m, and the steppe, growing even on the sea coast (López *et al.* 2013). The marked polymorphism made us think that the genetic variability behind it would explain both the morphological variability and the adaptability to different ecological niches.

In order to study genetic diversity and variability in the genus *Oxalis*, different techniques using molecular markers were used: to propose the origin of domestication and polyploidy in *Oxalis tuberosa* using ITS data (Emshwiller and Doyle 1998), AFLP (Tosto and Hopp 2000; Emshwiller *et al.* 2009); and *ncp*GS data (Emshwiller and Doyle 2002); to assess a model of bulb evolution using *nr*ITS and *trn*L–F (Oberlander *et al.* 2009); to propose the diversification of the American bulb-bearing *Oxalis* using ITS, *psb*J–*pet*A, *trn*L*–trn*F and *trn*T*–trn*L (Gardner *et al.* 2012); to explore distribution models and a dated phylogeny for Chilean *Oxalis* using *rbc*L, *trn*L–*trn*F, *psb*A–*trn*H, *trn*S–*trn*G and ITS (Heibl and Renner 2012); to propose a molecular phylogeny and chromosome evolution among *Oxalis* sections *Corniculatae* and *Ripariae* (Vaio *et al.* 2013, 2016); and to investigate genetic diversity and phenetic relationships within *Oxalis* species in Korea using RFLP (Huh and Choi 2014). However, there are no previous studies on the variability and genetic diversity in any species of section *Palmatifoliae* in which *O. laciniata* is included.

Here, we hypothesize that there is a pattern of differentiation within and among populations at the genetic level that can be test assessing the genetic structure and diversity. We also propose that the presence of exclusive haplotypes in populations reflects success in dissimilar environments, so we compare genetic patterns with geographical distribution. Finally, we intended to infer the phylogeographical structure using cpDNA haplotypes; and exam hypotheses of its survival *in situ* or in glacial refugia.

## Methods

### Sample collection and DNA extraction, PCR amplification

**Figure 1:**
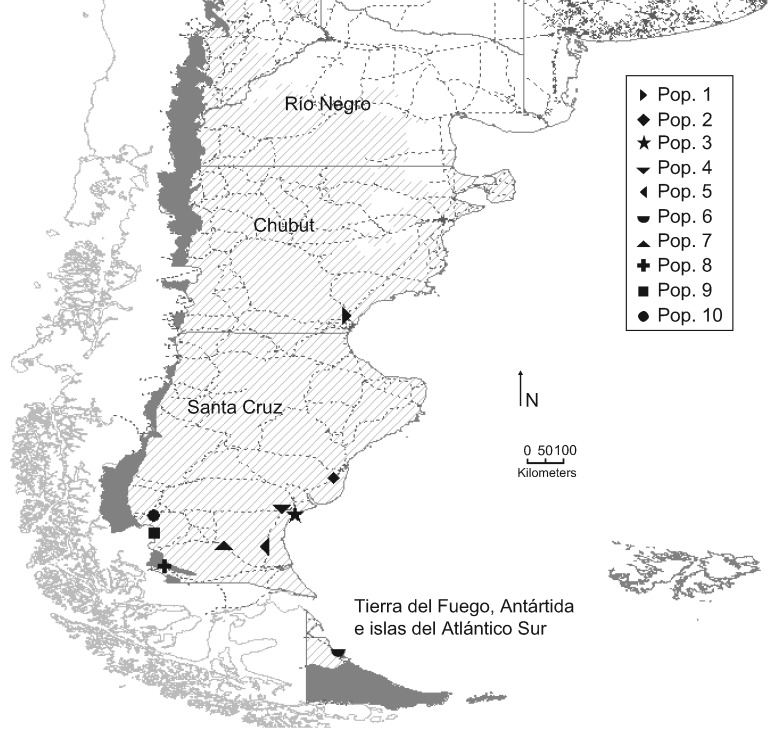
Geographical distribution of the analysed populations of *O. laciniata*. Dark gray indicates the High Andean ecoregion and striped gray the ecoregion of the Patagonian Steppe. For population abbreviations, see Table 1. Dotted lines indicate roads.

Individuals of *O. laciniata* were collected from ten natural populations in Patagonia Argentina, which are representative of the endemic distribution. A total of 67 individuals were analysed using ISSR markers, and 69 using cpDNA sequencing (Table 1; Fig. 1). Young leaf tissues from each sampled individuals were stored in plastic bags with silica gel.

**Table 1.** Sampling details of *O. laciniata* populations in the present study including: Population, collector, accession, sample size, in parenthesis sample size for ISSR, locality, longitude, latitude, altitude in meters above sea level.

Pop.	Colector	Accession	Sample Size	Locality			Lat.				Long.				Alt.
1	Zuloaga, F. O.	14644	4	Chubut	Escalante	Ruta Nacional 3, entre Ruta Provincial 27 y cruce con Ruta Provincial 37	45°	35′	38″	S	67°	38′	26″	W	625
2	Zuloaga, F. O.	14661	8 (6)	Santa Cruz	Corpen Aike	Ruta Nacional 3, al S de San Julián	49°	28′	36″	S	67°	55′	43″	W	145
3	López, A.	60	6 (8)	Santa Cruz	Corpen Aike	PN Monte León	50°	20′	51″	S	68°	53′	38″	W	30
4	López, A.	29	6	Santa Cruz	Corpen Aike	RN 3. Sur Comandante Piedrabuena	50°	14′	48″	S	69°	9′	2″	W	
5	López, A.	28	2	Santa Cruz	Güer Aike	Barranca Sur del Río Coyle	51°	9′	31,9″	S	69°	31′	7,8″	W	
6	López, A.	55	9	Tierra del Fuego	Río Grande	Cabo Domingo	53°	41′	28″	S	67°	50′	48″	W	1
7	Zuloaga, F. O.	14675	3 (4)	Santa Cruz	Güer Aike	Ruta Provincial 5, entre Las Horquetas y Esperanza	51°	7′	59″	S	70°	33′	12″	W	
8	López, A.	52	6 (7)	Santa Cruz	Güer Aike	Estancia Stag River	51°	36′	35″	S	71°	59′	23″	W	399
9	Zuloaga, F. O.	14679	14 (12)	Santa Cruz	Güer Aike	Estancia La Verdadera Argentina	50°	50′	2″	S	72°	14′	1″	W	960
10	Zuloaga, F. O.	14730	11 (9)	Santa Cruz	Lago Argentino	Cerro Huiliches	50°	22′	44″	S	72°	16′	4″	W	790

DNA extraction was performed following the modified CTAB protocol by Doyle and Doyle (1987), adapted for small amounts of plant material. Reactions were performed in a final volume of 25 µl. Each reaction contained ca. 50–100 ng of DNA, 1.5 units of Taq polymerase (Invitrogen life technologies São Paulo, Brazil), 1× PCR Buffer, 5-mM MgCl_2_, 0.2 µM of each primer and 0.025 mM of each dNTP. PCR amplifications were done as follows: an initial denaturation period at 94 °C for 5 min, followed by 35 cycles of denaturation at 94 °C for 30 s, annealing at 48 °C for 1 min, and extension at 72 °C for 1 min 30 s. Final extension at 72 °C for 6 min terminated the reactions. A negative control with no template was included for each series of amplifications to test for contamination. For ISSR markers, four positive controls were included in each PCR experiment to confirm band repetitively.

### ISSR markers

Nine ISSR primers were analysed (Table 2). PCR products were separated on 2 % agarose gels with 0.5× TAE electrophoresis buffer at 65 V for 60 min and stained with SyBr. The gels were exposed to ultraviolet light and photographed. The presence or absence of ISSR band was visually determined on the photographs. Control and a 100-bp ladder were used to compare the profiles among gels.

**Table 2.** Primers used for ISSR analysis, sequence and obtained number of bands per primer.

Primers	Sequences	No. bands
ISSR-1	(AC)8CA	12
ISSR-2	(AC)8GA	7
ISSR-3	(AC)8GT	7
ISSR-4	(AG)8T	6
ISSR-5	(ATG)6	8
ISSR-6	(CA)8G	8
ISSR-7	(CT)8G	7
ISSR-8	(GA)8YC	9
ISSR-9	(GT)8CG	9
Total bands		73

The amplified DNA fragments were scored as binary, and only those consistently reproducible bands were scored. Smeared and weak bands were excluded. Fragments of the same molecular weight were considered as the same locus. The resulting data matrix was analysed using GenAlEx 6.5 (Peakall and Smouse 2006). Genetic diversity within and among populations were measured by the percentage of polymorphic bands (PPB), the effective number of alleles (Ne), observed number of alleles (Na), Shannon’s information index (I) and frequency-down-weighted marker (DW). An unweighted pair-group method using arithmetic average (UPGMA) dendrogram and a Discriminant Analysis (DA) were constructed based on the matrix of Nei’s unbiased genetic distance using InfoStat 2013 (Di Rienzo *et al.* 2013). In addition, an analysis of molecular variance (AMOVA) procedure was performed. The variance components were tested statistically by nonparametric randomization tests using 999 permutations. Principal Coordinates Analysis (PCoA) was performed to explore relationships among population with Nei’s unbiased genetic distance matrix in FADM 1.30 software (Fingerprint Analysis with Missing Data, Schlüter and Harris 2006).

Structure 2.3.4 (Pritchard *et al.* 2000) was used to estimate the number of genetically distinct populations (*K*).The admixture model was used, which assumes correlated frequencies. The log-likelihood probability of the data was calculated for each possible *K* value from 1 to 13 using 5 runs of 15 × 10^6^ MCMC iterations following a burn-in period of 500 000 iterations. The best fit number of clusters was calculated according to Evanno *et al.* (2005) using Structure Harvester (Earl and vonHoldt 2012). This approach consists in plotting the second-order rate of change in ln Pr (*X*/*K*) for successive *K*s (referred to as Delta *K*) against a range of *K* values, and selecting the true *K* based on where the maximal value of this distribution occurs. The CLUMPP 1.1.2 (Jakobsson and Rosenberg 2007) was used to summarize the results of the best *K* value, and Distruct 1.1 (Rosenberg 2004) was used to create graphical outputs.

### cpDNA sequences

PCR products were sequenced by Macrogen, Inc. (Korea). Assembly and editing of sequences used the software Chromas Pro 1.34 (Technelysium Pty, Ltd). Sequences of *trn*H*-psb*A region were aligned using BioEdit 7.0.9.0 (Hall 1999). We also explored *trn*L-*trn*F and *rbc*L fragments, but no variation was absorbed by this marker. The alignment was then adjusted manually. All sequences were deposited in GenBank (*trn*H–*psb*A KY622055-KY622123).

We used ANeCA 1.2 (Panchal 2007) to calculate the TCS haplotype network taking into account the geographic distribution following the approach of Templeton *et al.* (1995) and Templeton (2004). The haplotype statistical parsimony network was constructed using TCS 1.21 (Clement *et al.* 2000). Clade (Dc) and nested clades (Dn) distances were estimated to assess association between the nested cladogram and geographical distances among sampled localities (Templeton *et al.* 1995) using the program Geodis 2.6 (Posada *et al.* 2000). Null distributions (i.e. under a hypothesis of no geographical association of clades and nested clades) for permutational contingency table test comparisons were generated from 10 000 Monte Carlo replications with 95 % confidence level.

In phylogenetic analyses, we included a sequence of each of the different haplotypes (16 sequences + 3 sequences as outgroup: *O. adenophylla* Cav., *O. enneaphylla* Cav. and *O. loricata* Dusén). Phylogenetic trees were calculated in TNT 1.1 (Goloboff *et al.* 2008), with all characters equally weighted. Heuristic searches were performed using 1000 random addition sequences and tree bisection-reconnection (TBR) branch swapping, saving ten trees per replicate. Branch support was assessed with 10 000 parsimony jackknifing (JK, Farris *et al.* 1996), using ten series of random addition sequences, swapped using TBR and holding two optimal trees per series. We also performed an uncorrelated-rates approach implemented in BEAST 1.8.0 (Drummond *et al.* 2012), which uses Bayesian Markov chain Monte Carlo (MCMC) runs to coestimate topology and node ages. We established as molecular model GTR+Γ using jModelTest 0.1.1 (Posada 2008) and constant coalescence size for the prior distribution. Since there are not fossils assignable to *Oxalis*, we calibrated the stem age calculated for *Oxalis* section *Palmatifoliae* in Heibl and Renner (2012), under strict clock.

For each MCMC analysis, we ran five independent chains for 10 million generations and assessed convergence and stationarity of each chain to the posterior distribution using TRACER 1.6 (Rambaut *et al.* 2014) and by plotting time series of the log posterior probability of sampled parameter values. After stationarity was achieved, we sampled each chain every 1000 steps.

### Distribution modelling

To estimate the distribution probabilities of species occurrence we used MaxEnt 3.3.3k (Phillips and Dudík 2008). We recorded the latitudes and longitudes from 14 localities of *O. laciniata*, covering its entire distribution. Environmental data with resolution of 2.5 arc min (5 km^2^) for current and past conditions were downloaded from WorldClim database (Hijmans *et al.* 2005a, http://www. worldclim.org/). We tested the two global climate models for the Last Glacial Maximum (LGM): the Community Climate System Model (CCSM) and the Model for Interdisciplinary Research on Climate (MIROC). We cut the grid layers with DivaGis 7.5.0.0 (Hijmans *et al.* 2005b) and selected our area of interest (from 76°17′24″ to 53°19′48″S, and from 56°19′48″ to 21°47′24″W). We evaluate Pearson correlation coefficient >0.75 to identify highly correlated variables. We used the following settings for the MaxEnt model: ten replicates with Boostrap, response curves, jackknife tests, logistic output format, random seed, random test percentage: 10 % to compute AUC (0.951), and to determine the threshold value we selected 10 percentile training presence. We used for the modelling 6 of the 19 bioclimatic variables available that were selected by comparing percent contribution values and jackknife plots (BIO: 1, 6, 10, 11, 13 and 16).

## Results

### Population variability and genetic patterns

From 67 individuals of 10 wild populations, 9 primers yielded 73 clearly identifiable and reproducible ISSR bands (Table 3). Of these bands 61.23 % were found to be polymorphic among all individuals. The total number of bands varied from 41 (in Pops 1 and 5) to 66 (in Pop 6). The effective number (Ne) of alleles varies from 1.232 in Pop 5 to 1.526 in Pop 2. The percentages of PPB were lower within Pop 5 (32.88 %) than within Pop 2 (79.45 %). Shannon’s information index (I) ranged from 0.442 to 0.199. The DW values ranged from 0.78 to 2.01. The Pops with the highest DW values were predominantly located in the High Andean region.

**Table 3.** Genetic diversity parameters for ISSR markers loci in *O. laciniata* including: Accession, no. of individuals (N), observed no. of alleles (Na), effective no. of alleles (Ne), no. of polymorphic loci, percentage of polymorphic loci (PPB), shannon’s information index (I), frequency-down-weighted marker (DW).

Accession	*N*	**Na** (± SE)	**Ne** (± SE)	No. of polymorphic loci	PPB (%)	***I*** (±SE)	DW
Pop. 1	4	0.959±0.108	1.275 ± 0.045	29	39.73	0.228±0.034	1.13
Pop. 2	6	1.603±0.093	1.526±0.044	58	79.45	0.442±0.030	1.29
Pop. 3	8	1.575±0.089	1.501±0.046	54	73.97	0.416±0.032	1.12
Pop. 4	6	1.562±0.083	1.438±0.045	50	68.49	0.373±0.033	1.46
Pop. 5	2	0.890±0.102	1.232±0.039	24	32.88	0.199±0.033	1.18
Pop. 6	9	1.548±0.078	1.466±0.049	47	64.38	0.374±0.035	0.89
Pop. 7	4	1.082±0.097	1.297±0.049	28	38.36	0.231±0.036	0.78
Pop. 8	7	1.507±0.090	1.501±0.048	49	67.12	0.402±0.035	1.60
Pop. 9	12	1.575±0.089	1.469±0.047	54	73.97	0.392±0.033	2.01
Pop. 10	9	1.575±0.089	1.435±0.044	54	7397	0.381±0.031	1.54

**Figure 2:**
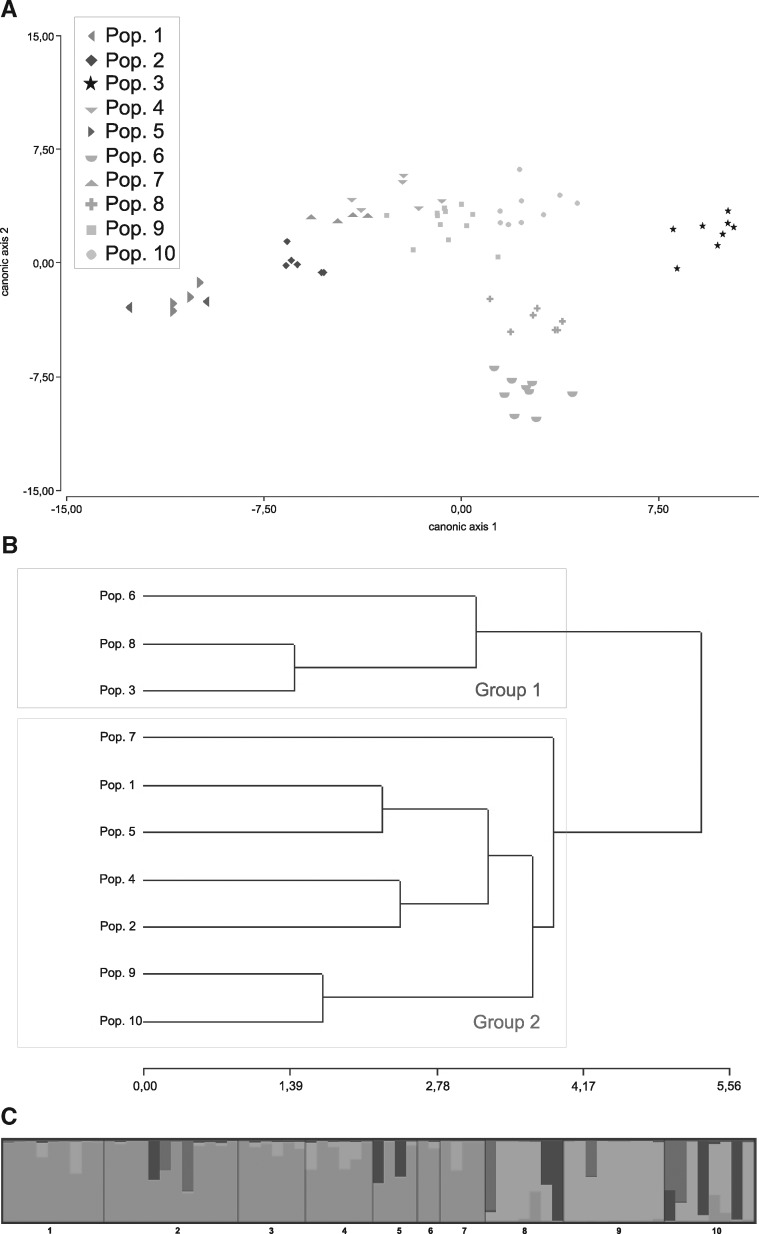
Population variability and genetic structure of *O. laciniata* based on ISSR markers. (**A**) DA of total ISSR data. LD1 and LD2 first and second discriminant axes, respectively. (**B**) UPGMA dendrogram based on Nei’s genetic distance, indicating the clustering relationships of sampled populations. (**C**) Clusters inferred with Structure at *K* = 4. Gray scale indicate individual estimated assignment fraction.

The DA (Fig. 2A) showed the grouping of the individuals belonging to the assigned populations. Although some overlapping was observed, the populations could clearly be identified as distinct groups.

The PCoA was also performed to determine the consistency of the differentiation of the individuals and among the populations. The analysis indicated that the first two principal coordinates accounted for 54.08 % of the total variation for individuals.

Dendrogram was constructed with Nei’s distance, by the UPGMA method (Fig. 2B). All the individuals analysed were separated into two large groups with a coefficient of 0.822. One of the groups includes populations 3, 6 and 8 (group 1) and the other one includes the remaining populations (group 2), with a coefficient of 5.3. The relationship established for all individuals from the ten populations in the cluster analysis based on ISSR maker were also found to be similar in the PCoA, as in the DA.

Structure results are shown in Fig. 2C. Individuals were assigned to four genetic groups, as suggested by Evanno *et al.* method, mostly corresponding with geographical location. Although the analysis assigned a *K* = 4, two clearly defined groups are evident. One would correspond to the populations of the Patagonia Steppe, and the other one to the populations of the High Andes ecoregion.

The AMOVA for the total marker data set in wild populations is shown in Table 4. According to these analyses, the genetic variation was 26 % between and 74 % within populations.

**Table 4.** Results of analysis of molecular variance (AMOVA) of ISSR data from 10 populations of *O. laciniata* including: Source of variance, degree of freedom (df), sume of squares (SS), mean of squares (MS), variance components, total variance (%), significance.

Source	df	SS	MS	Est. Var.	%
Among Pops.	9	322,557	35,840	3,803	26
Within Pops.	57	618,500	10,851	10,851	74
Total	66	941,057		14,654	100

### Analysis of haplotypes

**Figure 3:**
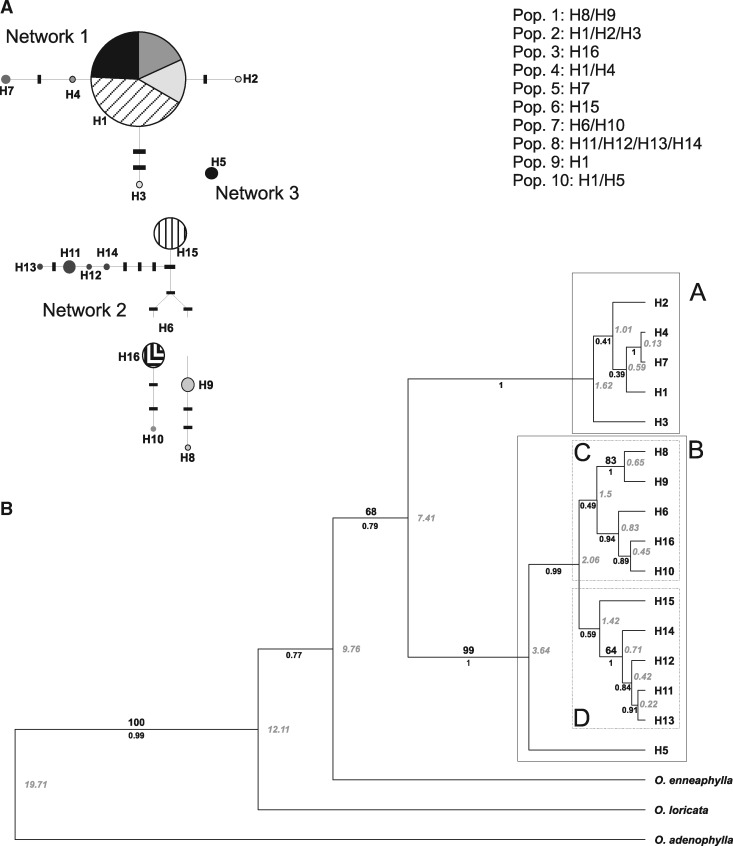
(**A**) Statistical parsimony network and resulting set of nested clades of the 16 chloroplast DNA haplotypes found. Circles indicate sampled haplotypes and solid bars are hypothetical haplotypes. (**B**) Consensus tree obtained from BEAST showing the inferred relationships within the haplotypes. Numbers on branches are JK support (above) and posterior probability values (below). Diversification time estimates, in Ma, are indicated in italics.

The total length of the aligned *trn*H*–psb*A region was 226 bp, of which 33 (13 %) were parsimony informative. Based on this variation, 16 different haplotypes were determined (H1–H16; Table 5). Statistical parsimony retrieved three different networks (Fig. 3A). In Network 1, the core haplotype (H1) is the most frequent and widespread considering the entire dataset. This haplotype is present in four populations (Pops: 2, 4, 9, 10) and represents 47.83 % of the total sampled individuals. Haplotype 1 is connected by one step to H4 (Pop 4), which is also connected by two steps with H7 (Pop 5). Haplotype 1 is also connected to H2 by two steps and with H3 by three steps; all three haplotypes are present in population 2. In Network 2, H15 (Pop 6) is connected with H14 (5 steps), H12 (6 steps), H11 (7 steps), and H13 (9 steps). All these latter four haplotypes are present in Pop 8. In addition H15 is connected with H6 (four steps, Pop 7), H16 (five steps, Pop 3), H10 (eight steps, Pop 7) and also with H9 (six steps) and H8 (nine steps), both present in Pop 1. At last, H5 (Pop 10) remains ungrouped constituting an exclusive Network.

**Table 5.** Haplotypes of *O. laciniata*. Haplotype, identification, frecuency (population).

Haplotype	ID	Frequency (population)
ALM08	H1	33 (6 Pop. 2, 5 Pop. 4, 14 Pop. 9, 8 Pop. 10)
ALM29	H2	1 (Pop. 2)
ALM26	H3	1 (Pop. 2)
ALM35	H4	1 (Pop. 4)
ALM38	H5	3 (Pop. 10)
ALM41	H6	2 (Pop. 7)
ALM44	H7	2 (Pop. 5)
ALM46	H8	1 (Pop. 1)
ALM47	H9	3 (Pop. 1)
JH1441	H10	1 (Pop. 7)
JH1894	H11	3 (Pop. 8)
JH1896	H12	1 (Pop. 8)
JH1898	H13	1 (Pop. 8)
JH1900	H14	1 (Pop. 8)
JH1904	H15	6 (Pop. 6)
JH1913	H16	6 (Pop. 3)

All the resulting trees obtained with both parsimony and Bayesian inferences were congruent. Parsimony resulted in one tree (length 64 steps, consistency index 0.703, and retention index 0.882). For discussion of the results, we adopted the hypothesis obtained from Bayesian analysis since it was the method with the highest resolution (Fig. 3B). From the tree topology inferred, two major clades can be identified: Clade A (PP = 1.00) including H1, H2, H3, H4 and H7; Clade B (JK = 99; PP = 1) including the remaining haplotypes. Clade B is subdivided into subclade C (PP = 0.49) including H6, H8, H9, H10 and H16; and subclade D (PP = 0.59) including H11, H12, H13, H14 and H15. Haplotype 5 does not appear to be related to the other haplotypes in clade B, which is consistent with the results of the haplotype network. In both analyses two clades with high support appear; one conformed by H8 and H9 (JK = 83; PP = 1), and the other by H11, H12, H13 and H14 (JK = 64; PP = 1).

The tree obtained by Bayesian inference was calibrated using a molecular clock with the proposed date of diversification for *Oxalis* Sect. *Palmatifoliae*. The estimated diversification times for the three main clades were the following: Clade A = 1.62; Clade C = 1.5 and Clade D = 1.42 Ma. The estimated times would place the diversification of *O. laciniata* in a period between the Andean orogeny (15–10 Ma) and Great Patagonian Glaciation (GPG), estimated of 1–1.2 Ma.

### Distribution modelling

**Figure 4:**
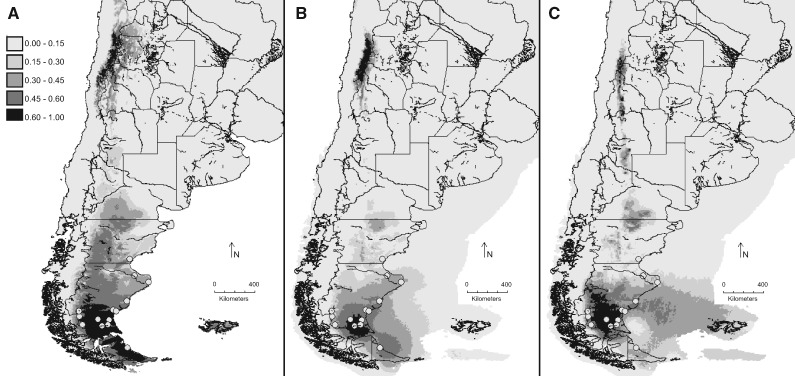
Habitat suitability in southern South America for *O. laciniata* calculated with MaxEnt. Darker greys indicate regions with a higher probability of species occurrence. Dots in light gray refer to point localities on which the models are based. Rivers are indicated in dark gray. (**A**) Present distribution model. LGM: (**B**) MIROC and (**C**) CCSM.

Present species distribution model for *O. laciniata* predicts that the area encompassing southern Argentina, from Chubut River down to the top tip of Tierra del Fuego e Islas del Atlántico Sur Province, provides the most suitable environment for this species (Fig. 4A), while the probability of occurrence ranges from low to moderate in the High Andes regions.

Potential Pleistocene geographic distribution in both global climate models, MIROC (Fig. 4B) and CCSM (Fig. 4C) showed some minor differences with the known current geographic distribution, showing a high occurrence of the species in a more restricted area. 

## Discussion

Here, we examine the genetic structure of *O. laciniata* across its entire distribution to test genetic variability. The presence of different haplotypes and the range-wide patterns of genetic diversity are supported by both ISSR markers and cpDNA Based on our findings we outline further prospects for integrative experimental and phylogeographical studies in *Oxalis*.

To evaluate population variability and to perform hypotheses about genetic patterns we also explored molecular markers data. The low level of polymorphism among populations (26 %) of *O. laciniata* revealed by ISSR markers are in agreement with previous studies performed in other species using other markers, for example 22 % using RAPD in *O. acetosella*, *O. corniculata*, *O. corymbosa, O. obtriangulata*, *O. stricta* (Huh and Choi 2014) and 10 % using AFLP in *O. tuberosa* and allies (Tosto and Hopp 2000). In addition, the Nei estimator results in an F_ST_ of less than 0.50, which indicates that more than a half of genetic differentiation occurred within populations. The major reason behind the low level of polymorphism among populations could be explained by the occurrence of high gene flow that may be facilitated by the presence of rivers and roads. Biological traits promote dispersing downstream and upstream along river valleys; while other factors associated with human activities facilitate its dispersal over high mountains, across river valleys and along the margins of the roads providing new scope for progressive invasions (Wang *et al.* 2011).

The genetic pattern of *O. laciniata* exhibits non-random organization of genetic variability suggesting a division of the populations into two primary groups. We propose that the larger group is composed of closely related populations, which not only share common alleles supported by ISSR results, but also the genealogy of chloroplasts. The other group includes populations that are more isolated, and our hypothesis is that their grouping is due not to sharing common alleles but to having private alleles. High DW values are expected in long-term isolated populations, which accumulate population-specific mutations that are quickly fixed but not dispersed to other populations (Daneck *et al.* 2015). DW values of populations 3 and 8 are in agreement with this hypothesis.

To infer the origin of populations from their present distribution pattern we used cpDNA data to test hypotheses about both, glacial refugia and *in situ* survival for *O. laciniata*. According to our findings, one of the main glacial refugia would be located in the Gulf of San Julián in the SE, which is in line with the proposal of Nicola *et al.* (2014) and supported by the presence of haplotype diversity and also by the high DW value. Second refugia was situated in the SW, in the area of Ea. Stag River, La Verdadera Argentina, Co. Huiliches and surroundings, in agreement with Cosacov *et al.* (2010). We can speculate about a chloroplast capture event that caused the appearance of the distant chloroplast type in population 8. If we take into account the sympatry of *O. laciniata* and two other *Oxalis* species of section *Palmatifoliae*, *O. loricata* and *O. enneaphylla* in the same locality (Ea. Stag River), a hybridization process could be possible. Nevertheless, further detailed study in this region, using nuclear molecular data, including all the other taxa of Sect. *Palmatifoliae* is needed to clarify this finding more precisely. The refugia in the SE is supported by both the cpDNA and ISSR results, whereas the refugia in the SW is strongly supported by the ISSR data but not by the cpDNA data since H1 is present in 33 individuals belonging to 4 different populations. The results suggest that populations with low DW values may have survived during the last glaciations in the same place as they currently inhabit, supporting the hypothesis of *in situ* diversification.

The analysis of population 7 allows us to infer that the centre of diversification of the species could have been in the steppe (Las Horquetas y Esperanza localities), where haplotypes H6 and H10 are present. If we consider the position in the network and the geographical location of these haplotypes, we can propose that they would be ancestral with respect to H16 (Pop 3), H8 and H9 (Pop 1), H15 (Pop 6), and that the most derived haplotypes would be H11, H12, H13 and H14, all present in the population 8. In contrast, population 10, located in Co. Huiliches, also holds an exclusive haplotype (H5) that does not appear to be linked to any of the others, constituting an isolated network by its own.

Our results of distribution modelling are in agreement with the present distribution of all the species of *Oxalis* Sect. *Palmatifoliae*. The locality data obtained from herbarium material of *O. laciniata* coincide with the area that predicts our distribution modelling for the species, ranging from the Chubut River to the tip of Tierra del Fuego e Islas del Atlántico Sur Province. Furthermore, the probability of being found in the High Andes regions of the North of Argentina and Chile showed a new suitable habitat for this species. Outstandingly, the highest probability of occurrence, indicating the most suitable environment, is in correspondence with our proposed centre of diversification.

Glacial climate fluctuations had a substantial impact on the diversification, distribution and demography of the Patagonian species. A scenario of multiple periglacial Pleistocene refugia and subsequent multiple recolonization routes, from eastern Patagonia to the Andean flanks, were proposed by several authors to explain the phylogeographical patterns of the present day distribution (Moritz 2002; Morando *et al.* 2007; Cosacov *et al.* 2010; Sede *et al.* 2012; Nicola *et al.* 2014; Morello and Sede 2016). In the modelling of the past scenario, there is a greater probability of occurrence coincident with the centre of the current geographical distribution, which is also proposed as the centre of diversification of the species. The climatic variables that were used correspond to the LGM period, and not to the GPG (bioclimatic layers are not available), which is where the diversification of the haplotypes would have occurred according to the dating obtained in this study. According to the hypothesis that suggests that the Patagonian biota has been shaped by two important geological and climatic events, the Andean orogeny during the Miocene (15–10 Ma) and Pleistocene glaciations (Sede *et al.* 2012), our results situate the diversification of *O. laciniata* within this period. Therefore, these results are preliminary and require more detailed studies of the biological, geological and climatic variables of the past.

The relatively high level of genetic diversity in *O. laciniata* compared with other *Oxalis* species such as *O. tuberosa* and allies (Tosto and Hopp 2000; Emshwiller *et al.* 2009) and *O. acetosella*, *O. corniculata*, *O. corymbosa*, *O. obtriangulata*, *O. stricta* (Huh and Choi 2014) could be a result of many factors such as breeding system, seed dispersal, genetic drift, evolutionary history as well as life form. In general, long-lived and out crossing species tend to be more genetically diverse (Hamrick and Godt 1996). *O. laciniata* is a perennial herb with a complex breeding system that presents tristily (Gardner *et al.* 2012; López *et al.* 2013). All populations analysed in this study were sampled including all the three flower morphs (longistyles, brevistyles and mesostyles), and can contribute to explain the high genetic diversity.

We propose the following causes for high genetic diversity in *O. laciniata*. Firstly, mating systems have been postulated to be one of the most important factors that determine the genetic diversity in plant species. Second, self-incompatibility is believed important in the maintenance of the high amount of genetic variability of species, and finally, the geographic distribution can affect the degree of genetic diversity of a species, the species with wide distribution being those that have greater degrees of variability.

## Conclusions

Here, we provide the first range-wide assessment of haplotype diversity and genetic structure of *O. laciniata*, a representative of *Oxalis* which is an important emerging model in evolutionary biology and phylogenetic studies. Our conclusions are: (i) the genetic structure revealed low level of polymorphism among populations and high genetic variation within populations; (ii) the phylogeographical pattern demonstrated non-random organization of genetic variability and two distinctly groups could be distinguished; (iii) two refugia are proposed: one situated in the SE that also holds the more ancestral haplotypes; and the second one situated in the SW; (iv) we also proposed *in situ* diversification hypothesis for the populations located in the steppe; (v) the centre of diversification is in coincidence with the centre of the distribution; (vi) distribution modelling shows a strong correspondence with the distribution of the species but also suggests the possibility of occurrence in the High Andes regions.

We proposed that some of the reasons behind the high genetic variation of *O. laciniata* are (i) outcrossing facilitated by tristyly that includes both a complex mating system and self-incompatibility; (ii) widespread distribution including heterogeneous environments (High Andean and Steppe ecoregions). The intriguing question whether such genetic variation might be linked with incipient polyploid speciation should also be tested by assessing the strength of reproductive isolation among the spatially, genetically and ecologically divergent lineages. Further investigations are necessary to test whether genetic structure and genetic variation within and among populations resulted from random population genetic processes or are the result of historical processes occurring in the Patagonia.

## Sources of Funding

A.L. acknowledges the National Research Council of Argentina (CONICET) as researcher and M.G.B. as postdoctoral fellowship holder. The project was supported by Préstamo BID PICT2013-0291, and PIP-112201301 00 124CO. 

## Contributions by the Authors

A.L. conceived the idea, collected the samples and performed the laboratory work; M.G.B. performed the molecular and statistical analyses; A.L. and M.G.B. analysed the data and led the writing.

## Conflict of Interest Statement

None declared.
